# *β*-Arrestin1 inhibits chemotherapy-induced intestinal stem cell apoptosis and mucositis

**DOI:** 10.1038/cddis.2016.136

**Published:** 2016-05-19

**Authors:** Y Zhan, C Xu, Z Liu, Y Yang, S Tan, Y Yang, J Jiang, H Liu, J Chen, B Wu

**Affiliations:** 1 Department of Gastroenterology, The Third Affiliated Hospital of Sun Yat-Sen University, Guangzhou, Guangdong Province, China; 2 Department of Gynecology and Obstetrics, The Third Affiliated Hospital of Sun Yat-Sen University, Guangzhou, Guangdong Province, China; 3 Department of Pathology, The Third Affiliated Hospital of Sun Yat-Sen University, Guangzhou, Guangdong Province, China

## Abstract

The mechanism of chemotherapy-induced gastrointestinal (GI) syndrome (CIGIS) is still controversial, and it is unclear whether chemotherapy induces intestinal stem cell (ISC) apoptosis. *β*-Arrestins are regulators and mediators of G protein-coupled receptor signaling in cell apoptosis, division and growth. In this study, we aimed to investigate whether chemotherapy induces ISC apoptosis to contribute to mucositis in CIGIS and whether *β*-arrestin1 (*β*-arr1) is involved in this apoptosis. Different chemotherapeutic agents were used to generate a CIGIS model. *Lgr5-EGFP-IRES-creERT2*^*+/−*^ knock-in mice were used as a CIGIS model to investigate ISC apoptosis. *β-arr1* knockout mice were used to determine whether *β-arr1* is involved in the apoptosis in CIGIS. Intestinal histology was performed, the ISC apoptosis was analyzed and the mucosal barrier was examined. The effects of *β-arr1* in apoptosis were investigated in the samples from humans and mice as well as in cell lines. Here, we demonstrate that chemotherapy induced intestinal mucositis by promoting crypt cell apoptosis, especially in Lgr5+ stem cells and Paneth cells but not in goblet cells, epithelial cells or vascular endothelial cells. Furthermore, *β-arr1* deficiency exacerbated the Lgr5+ stem cell apoptosis, but not Paneth cell apoptosis, in CIGIS. In addition, the data showed that *β*-arr1 reduced the chemotherapy-induced Lgr5+ stem cell apoptosis by inhibiting endoplasmic reticulum stress-mediated mitochondrial apoptotic signaling. Our study indicates that *β*-arr1 inhibits chemotherapy-induced ISC apoptosis to alleviate intestinal mucositis in CIGIS.

Gastrointestinal (GI) toxicity is the main limitation in chemotherapy and results in damage to the small intestine and complications that are known as GI syndrome.^1^ However, the cellular targets and underlying mechanisms of chemotherapy-induced GI syndrome (CIGIS) are still very controversial. Some believe that damage to the endothelial cells induces mucosal ischemia, which results in epithelial dysfunction.^2^ Others proposed that the decrease in goblet cells alters mucin secretion and intestinal flora, which leads to intestinal mucositis.^3^ Different chemotherapy agents have been shown to target particular cell positions within the crypt hierarchy. Recent studies demonstrated that progenitor cell apoptosis was induced by chemotherapeutic drugs.^4^ A study by our group found that chemotherapy increased intestinal crypt cell apoptosis and inhibited proliferation.^5^ However, no studies have explained the relationship between intestinal stem cells (ISCs) and CIGIS. Increasing evidence has indicated that the crypt base columnar (CBC) cells that express leucine-rich repeat-containing G protein-coupled receptor 5 (Lgr5) are ISCs.^6, 7^ Nevertheless, because these long slender CBC cells are in close proximity to Paneth cells, which are much more visually prominent, and because there were no unique markers for CBC cells until the *Lgr5* knock-in mouse model was developed,^8^ it is still unclear whether the CBC cells are involved in CIGIS. In this study, we found that Lgr5+ CBC cells undergo apoptosis after chemotherapy.

Several signaling pathways have been shown to regulate chemotherapy-induced apoptosis in the crypt cells, including the p53 pathway, which was identified in our recent study.^5^ *β*-arrestin1 (*β*-arr1) was originally classified as a G protein-coupled receptor (GPCR) but was later shown to have much broader and more versatile roles. *β*-arr1 is involved in maintaining cellular homeostasis^9^ and has an important role in the pathogenesis of various diseases, including sepsis,^10^ cerebral ischemia^11^ and asthma.^12^ Based on a previous study, *β*-arr1 facilitated Akt-mediated activation of MDM2 (human murine double minute 2, the E3 ligase for p53) and also promoted MDM2 binding to, and degradation of, p53 by acting as a molecular scaffold.^13^ Based on these results, we hypothesized that inhibition of *β*-arr1 may aggravate chemotherapy-induced apoptosis via activation of p53. The activation of p53 then induces p53-dependent endoplasmic reticulum (ER) stress, which promotes cell apoptosis and inhibits the stemness of stem cells.^14^ Moreover, a recent report suggested that induction of ER stress decreases stem cells via differentiation, resulting in a loss of stem cells in the intestinal crypts.^15^ Here, our results show that a decrease in stem cells after chemotherapy may be linked to ER stress.

In this study, *β*-arr1 reduced the chemotherapy-induced Lgr5+ stem cell apoptosis by inhibiting ER stress-mediated mitochondrial apoptotic signaling to alleviate intestinal mucositis in CIGIS.

## Results

### Chemotherapy-induced intestinal injury and mucositis

**Figure 1:**
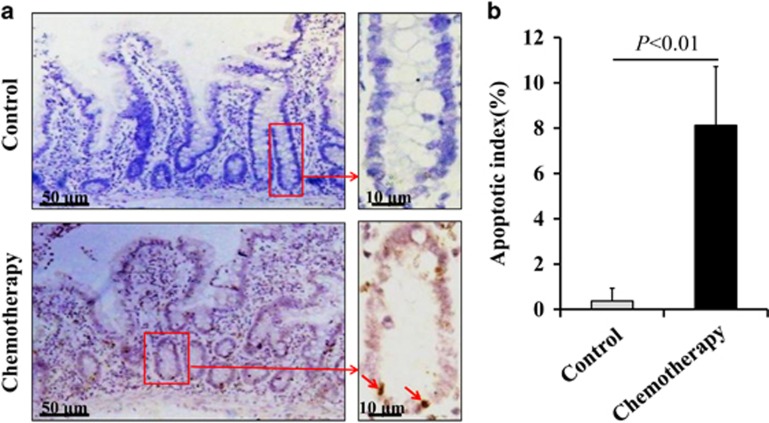
Chemotherapy-induced intestinal crypt cell apoptosis in patients. (**a**) Apoptosis was assessed by TUNEL staining (brown), and the arrows indicate TUNEL-positive cells. Partial apoptotic cells found in the stem cell niche in chemotherapy patients. (**b**) Human intestinal crypt apoptotic index measured by TUNEL staining was increased after chemotherapy. Values are presented as the mean±S.D., *n*=3 in each group

**Figure 2:**
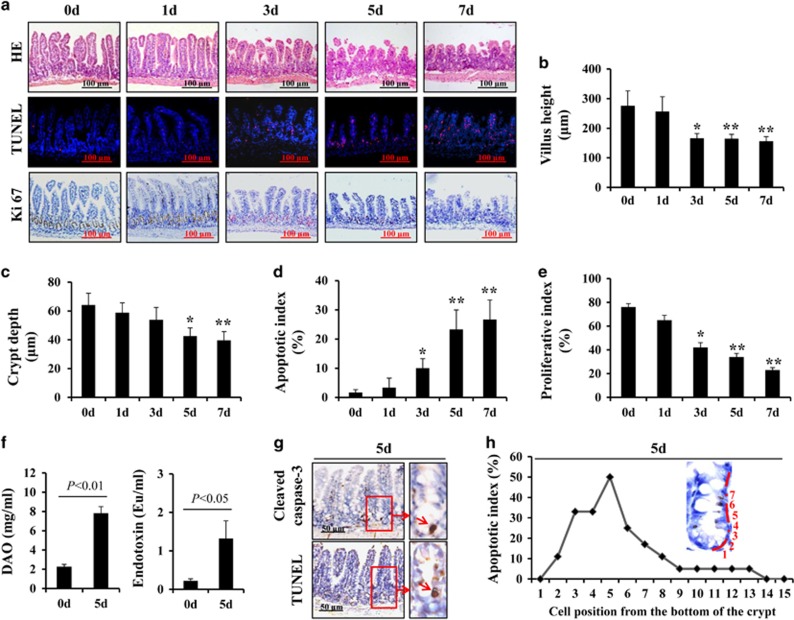
5-FU treatment induced intestinal mucositis. (**a**) Morphological changes were evaluated by H&E staining, apoptosis of intestinal crypt cells was assessed by TUNEL staining (red) and proliferation of intestinal crypt cells was measured by Ki67 staining (brown), magnification × 200. (**b**) Villus height in the small intestine decreased with increasing therapeutic dose of 5-FU. Values are shown as the mean±S.D., *n*=4 in each group. *P<0.05, **P<0.01, compared with the 0-day control. (**c**) Crypt depth in the small intestine was decreased with increasing therapeutic dose of 5-FU. Values are shown as the mean±S.D., *n*=4 in each group. *P<0.05, **P<0.01, compared with the 0-day controls. (**d**) Intestinal crypt proliferative index measured by Ki67 staining was reduced after 5-FU treatment. Values are shown as the mean±S.D., *n*=4 in each group. *P<0.05, **P<0.01, compared with the 0-day control. (**e**) Intestinal crypt apoptotic index measured by TUNEL staining was increased after 5-FU treatment. Values are shown as the mean±S.D., *n*=4 in each group. *P<0.05, **P<0.01, compared with the 0-day control. (**f**) Blood DAO levels were examined by ELISA to estimate intestinal permeability. Blood endotoxin levels were examined by ELISA to estimate intestinal bacterial translocation. Values are shown as the mean±S.D., *n*=6 in each group. (**g**) Caspase-3 staining and TUNEL staining in the sections. (**h**) Cells in the intestinal crypt zone were induced to apoptosis after 5 days of 5-FU treatment

Our study showed that intestinal crypt cells underwent apoptosis in chemotherapy patients with CIGIS but not in non-chemotherapy patients (Figure 1). To elucidate the pathogenesis of crypt apoptosis, we established animal models with intraperitoneal (i.p.) injections of 5-fluorouracil (5-FU) at a dose of 75 mg/kg/day every 24 h for 1, 3, 5 and 7 days. CIGIS worsened with increasing total dose of 5-FU. Histological evaluation revealed that 5-FU treatment resulted in significant intestinal mucosal injury (Figure 2a). Meanwhile, a mild switch in the apoptotic area was observed with prolonged 5-FU treatment (Figure 2a). The dynamic changes in apoptotic cells should be addressed in further studies. The small intestinal villus height decreased, and crypt depth was reduced after 5-FU treatment for 5 days compared with vehicle-treated mice (Figures 2b and c). Following 5-FU treatment, the apoptosis in the intestinal mucosa gradually increased (Figures 2a and d), and the proliferation of the intestinal crypt cells was gradually inhibited (Figures 2a and e). As a result, the intestinal permeability was significantly higher, and bacterial translocation evidently increased (Figure 2f), which are the characteristics of intestinal mucositis.^16^ Furthermore, we observed that apoptosis was centered in the bottom of the crypts, especially positions 3–6 of the crypts, where the apoptotic index ranged from 25 to 50% (Figures 2g and h).

### Chemotherapy-induced ISC and Paneth cell apoptosis

**Figure 3:**
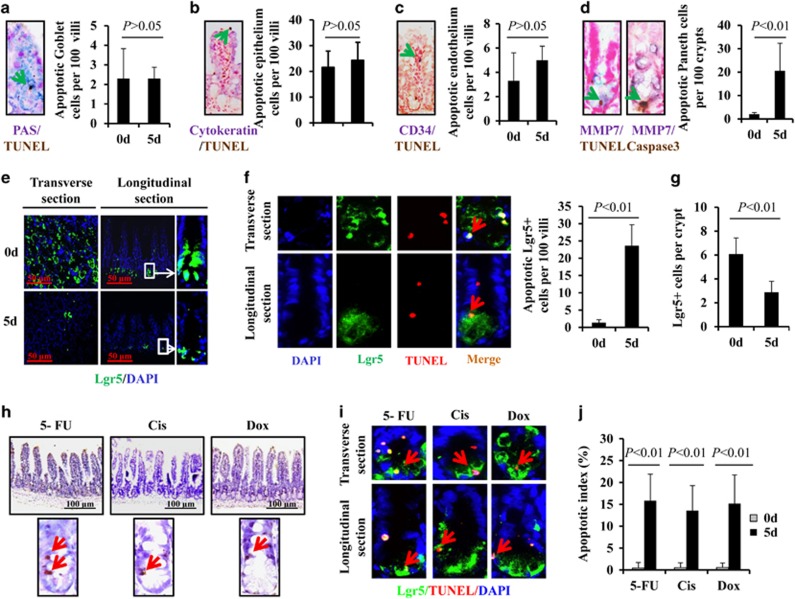
Chemotherapy-induced Paneth cell and Lgr5+ stem cell apoptosis. (**a**) Section double stained with TUNEL (brown) and PAS (purple, labeled goblet cells). The arrow indicates double-positive cells, magnification × 400. (**b**) Section stained with TUNEL (brown) and anti-cytokeratin (purple, labeled epithelial cells). The arrow indicates double-positive cells, magnification × 400. (**c**) Section stained with TUNEL (brown) and anti-CD34 (purple, labeled endothelial cells). The arrow indicates double-positive cells, magnification × 400. (**d**) Section stained with TUNEL (brown) and anti-MMP7 (purple, labeled Paneth cells) or anti-caspase-3 (brown) and anti-MMP7. Arrows indicate double-positive cells, magnification × 400. Values are shown as the mean±S.D., *n*=4 in each group. (**e**) 5-FU treatment decreased intestinal Lgr5+ stem cells. (**f**) 5-FU induced apoptosis in Lgr5+ stem cells. Sections were stained with TUNEL (red) and anti-EGFP (green, labeled Lgr5+ stem cells), magnification × 400. Values are expressed as the mean±S.D., *n*=4 in each group. (**g**) After 5 days of administration, 5-FU significantly decreased the quantity of Lgr5+ stem cells. Values are shown as the mean±S.D., *n*=4 in each group. (**h**) Treatment with different chemotherapeutic agents induced intestinal crypt cell apoptosis. Sections were stained with TUNEL. (**i**) Treatment with different chemotherapeutic agents induced Lgr5+ stem cell apoptosis. Sections were double stained by anti-EGFP (Lgr5) and TUNEL. Red arrows indicate the double-positive cells. (**j**) The apoptotic index was increased by treatment with different chemotherapeutic agents. Values are shown as the mean±S.D., *n*=4 in each group

To investigate apoptotic target cells in CIGIS, TUNEL staining was used to identify apoptosis, and several distinct cell markers, including periodic acid–Schiff (PAS), cytokeratin, CD34 and MMP7, were used to assess different types of cells in the crypts. Double immunostaining showed that apoptosis of goblet cells (PSA positive), epithelial cells (cytokeratin positive) and vascular endothelial cells (CD34 positive) was rarely observed after chemotherapy (Figures 3a and d). However, apoptosis of Paneth cells (MMP7 positive) was induced (Figure 3d). After 5-FU treatment for 5 days, an average of 21 apoptotic Paneth cells in every 100 crypts was observed, which was substantially increased compared with 5-FU treatment for 0 days (Figure 3d). To examine the response of stem cells following 5-FU treatment, *Lgr5* knock-in mice were used to evaluate ISC apoptosis. Lineage tracing indicated that Lgr5-expressing cells at the base of the crypt can function as stem cells for all four epithelial lineages.^8^ Our data revealed that Lgr5+ stem cells were notably reduced after 5-FU treatment for 5 days (Figure 3e). Double immunostaining confirmed that 5-FU-induced apoptosis led to a reduction in Lgr5+ stem cells (Figures 3f and g). These results show that 5-FU induces marked apoptosis in both Paneth cells and Lgr5+ stem cells.

To elucidate the effects of chemotherapy in CIGIS, other two classical chemotherapeutic agents (cisplatin (Cis) and doxorubicin (Dox)) were used in the study. The results showed that, similar to 5-FU, apoptosis was also observed in the bottom of the crypts after Cis and Dox treatment for 5 days, and apoptosis was predominantly observed in Lgr5+ stem cells (Figures 3h and i). The apoptotic index confirmed that apoptosis of Lgr5+ stem cells was substantially increased in chemotherapy-induced CIGIS (Figure 3j). Taken together, the results strongly suggest that apoptosis of Lgr5+ stem cell contributes to CIGIS.

### *β*-arr1 deficiency aggravated chemotherapy-induced intestinal crypt cells apoptosis

**Figure 4:**
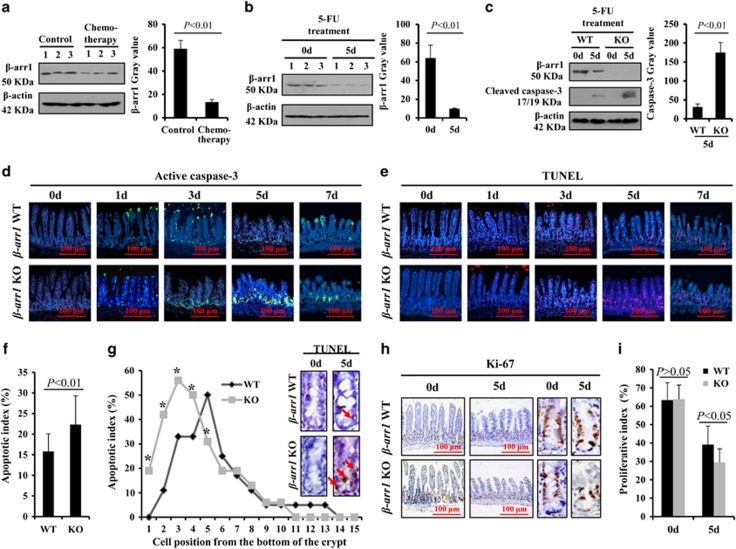
*β-arr1* deficiency aggravated apoptosis in the bottom of the intestinal crypt after 5-FU treatment. (**a**) *β*-arr1 was inhibited in patients after chemotherapy as indicated by western blotting (*P*<0.01). (**b**) The protein expression of *β*-arr1 in the mouse small intestinal mucosa was detected by western blotting after 5-FU treatment, and *β*-actin was used as a loading control. *β*-arr1 in the mice significantly decreased after chemotherapy (*P*<0.01). (**c**) The protein expression of *β*-arr1 and cleaved caspase-3 was analyzed by western blotting in the small intestinal mucosal of *β-arr1* WT and KO mice after 5-FU treatment. After 5 days of 5-FU treatment, cleaved caspase-3 was more evidently enhanced in KO mice than in WT mice (*P*<0.01). (**d**) Intestinal cleaved caspase-3 (green) was stained by immunofluorescence, magnification × 200. (**e**) Intestinal TUNEL (red) staining, magnification × 200. (**f**) The apoptotic index was determined after 5-FU treatment for 5 days. Values are expressed as the mean±S.D., *n*=4 in each group. (**g**) TUNEL staining displayed the apoptotic cells located in the crypt bottom after 5-FU treatment for 5 days. Arrows indicate apoptotic cells, magnification × 400. The apoptotic cells were predominantly located in positions 3–5 in the crypts in WT mice but positions 2–4 in KO mice. Values are expressed as the mean±S.D. in each cell position, *n*=4 in each group, *P<0.01. (**h**) *β-arr1* deficiency inhibited Ki67 expression in CIGIS. (**i**) The Ki67 index was distinctly decreased after 5-FU treatment in the KO mice compared with WT mice

After chemotherapy, *β*-arr1 in the intestinal mucosa was significantly downregulated in patients (Figure 4a). Thus, to verify the role of *β*-arr1 in CIGIS, *β-arr1* wild-type (WT) and knockout (KO) mice were used. Intestinal mucosal *β*-arr1 expression was significantly reduced by 5-FU treatment, and *β*-arr1 deficiency evidently enhanced cleaved caspase-3 expression compared with WT mice (Figures 4b and c). Furthermore, TUNEL staining and immunofluorescence staining of active caspase-3 confirmed that the apoptosis in *β-arr1* KO mice was notably increased following 5-FU treatment (Figures 4d–f). The apoptosis was principally located at the bottom of the crypts, especially positions 3–5 of the crypts, and *β-arr1* deficiency markedly increased the apoptosis in positions 2–4 of the crypts (Figure 4g). In addition, *β-arr1* deficiency aggravated the inhibition of crypt cell proliferation, and the proliferative index was lower in the *β-arr1* KO mice than the *β-arr1* WT mice (Figures 4h and i).

### *β*-arr1 deficiency increased chemotherapy-induced ISC apoptosis

**Figure 5:**
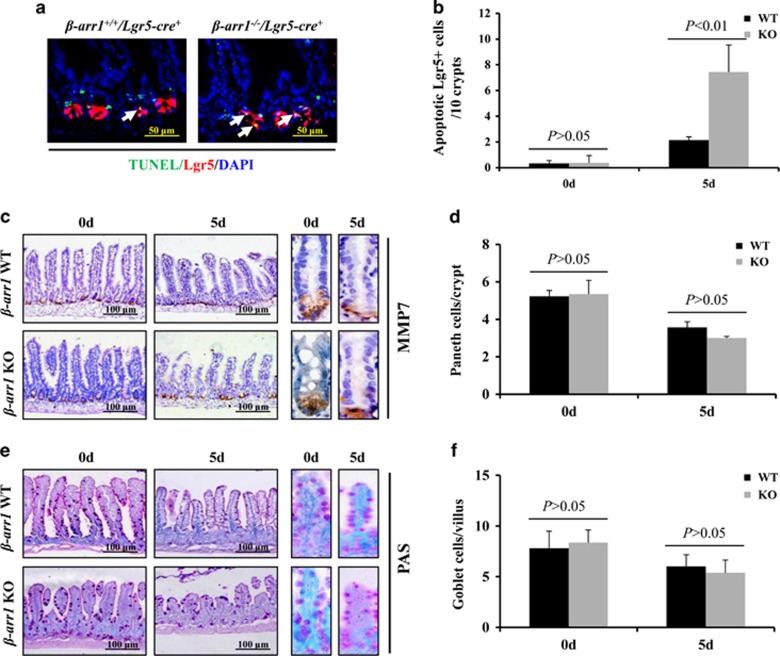
*β-arr1* deficiency increased ISC apoptosis after 5-FU treatment. (**a**) Intestinal sections with the indicated genotypes were subjected to TUNEL (red) and EGFP (green, to detect Lgr5+ cells) staining. White arrows indicate double-positive signals. (**b**) Apoptotic Lgr5+ stem cells were counted in every 10 crypts after 5-FU treatment for 5 days. Values are shown as the mean±S.D., *n*=4 in each group. (**c**) Sections were stained for MMP7 (brown) to label Paneth cells. (**d**) Paneth cells were reduced after 5-FU treatment. However, *β*-arr1 deficiency did not affect the Paneth cells. Values are shown as the mean±S.D., *n*=4 in each group. (**e**) Sections were stained with PAS (magenta) to label goblet cells. (**f**) Goblet cells were not significantly reduced after 5-FU treatment, and *β*-arr1 deficiency did not affect the goblet cells. Values are shown as the mean±S.D., *n*=4 in each group

To confirm that post-chemotherapy intestinal crypt apoptosis occurs in the stem cells, we crossed *β-arr1*^*+/−*^ mice to *Lgr5-cre*^*+/−*^ mice, and obtained *β-arr1*^*+/+*^*/Lgr5-cre*^*+/−*^ mice and *β-arr1*^*−/−*^*/Lgr5-cre*^*+/−*^ mice. TUNEL and EGFP (Lgr5) co-staining showed that apoptosis in Lgr5+ stem cells was induced, and the apoptosis of Lgr5+ stem cells was notably increased in *β-arr1*^*−/−*^*/Lgr5-cre*^*+/−*^ mice compared with the *β-arr1*^*+/+*^*/Lgr5-cre*^*+/−*^ mice at 5 days after 5-FU treatment (Figures 5a and b). However, the apoptotic signal of Lgr5+ stem cells was low at 0 days of 5-FU treatment (data not shown).

In addition to Lgr5+ stem cells, the apoptosis of Paneth cells was also observed after 5-FU treatment for 5 days (Figure 3d). To investigate the effects of Paneth cells in CIGIS, Paneth cells were labeled by MMP7 using immunohistochemical staining, and the results showed that although chemotherapy induced apoptosis of the Paneth cells, *β-arr1* deficiency did not reduce the number of Paneth cells after 5-FU treatment for 5 days compared with WT mice (Figures 5c and d). To investigate the effect of goblet cells in CIGIS, goblet cells were labeled by PAS staining, and the results also showed that *β-arr1* deficiency did not affect the number of goblet cells after 5-FU treatment for 5 days compared with WT mice (Figures 5e and f).

### Deletion of *β*-arr1 enhanced intestinal injury, permeability, bacterial translocation, and reduced mortality in mice after 5-FU treatment

**Figure 6:**
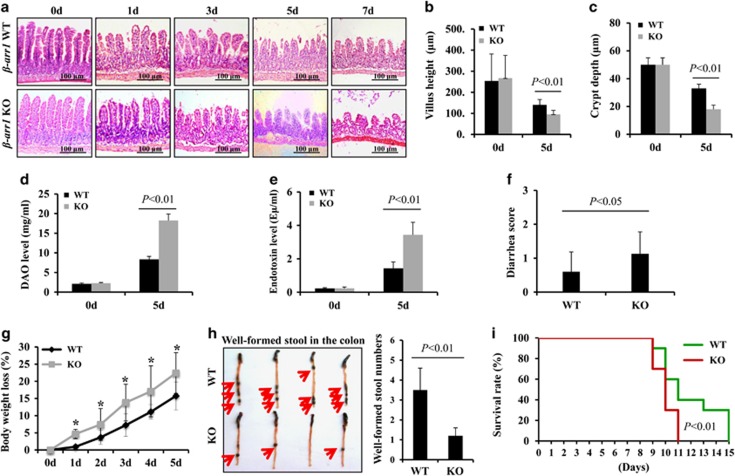
Deletion of *β-arr1* decreased the survival rate of mice following 5-FU treatment. (**a**) Intestinal sections were stained with H&E. (**b**) Villus height was assessed. Values are shown as the mean±S.D., *n*=4 in each group. (**c**) Crypt depth was evaluated. Values are shown as the mean±S.D., *n*=4 in each group. (**d**) Intestinal permeability following 5-FU treatment was evaluated by measuring serum DAO (mg/ml). Values are shown as the mean±S.D., *n*=4 in each group. (**e**) Intestinal bacterial translocation following 5-FU treatment was measured by serum endotoxin (E*μ*/ml). Values are shown as the mean±S.D., *n*=4 in each group. (**f**) Average diarrhea score in mice exposed to 5-FU, *n*=10 in each group. (**g**) Body weight loss in mice exposed to 5-FU, *n*=10 in each group, *P<0.01. (**h**) Well-formed stool in the colon. After 5-FU treatment for 5 days, the mice were killed, and the colon was harvested. Loose, yellow content in the lumen is indicative of poor stool formation or diarrhea, whereas solid, dark, granulated contents are well-formed stools (indicated by red arrows). (**i**) Survival curves were determined after mice were subjected to 5-FU treatment (50 *μ*g/kg/day). The difference in survival rate between *β*-arr1 WT and KO mice was significant (*P*<0.01)

To explore the effects of *β*-arr1 in CIGIS, the morphological changes in the intestinal mucosa were observed in both *β-arr1* WT mice and KO mice following 5-FU treatment. Progressive reductions in the height of the villus and the depth of the crypt were found in both *β-arr1* WT and *β-arr1* KO mice; however, the reductions were more severe in *β-arr1* KO mice than in *β-arr1* WT mice (Figure 6a). After 5 days of 5-FU treatment, the villus height was significantly smaller, and the crypt depth was also notably reduced in *β-arr1* KO mice compared with WT mice (Figures 6b and c). In addition, the intestinal permeability was assessed by measuring the systemic plasma concentration of diamine oxidase (DAO), and the data showed that the intestinal permeability was significantly increased in *β-arr1* KO mice compared with WT mice (Figure 6d) after 5-FU stimulation. In addition, the intestinal bacterial translocation was evaluated by examining endotoxin levels in the blood, and the data showed that the endotoxin level was doubled in *β-arr1* KO mice compared with WT mice (Figure 6e). These data indicate that deletion of *β-arr1* enhanced chemotherapy-induced intestinal injury and intestinal bacterial translocation. Furthermore, the intestinal function, the intestinal morphology and the survival rate were analyzed in mice administered 5-FU at a dose of 50 mg/kg/day i.p. Body weight, diarrhea and survival of mice were assessed once a day and recorded. The data showed that diarrhea and body weight loss significantly increased in *β-arr1* KO mice compared with WT mice (Figures 6f and g). Moreover, the mice were killed after 5 days of 5-FU injection to observe the well-formed stool in the colon, and a large number of well-formed stool were observed in WT mice but not in *β-arr1* KO mice (Figure 6h). The survival rate of the WT mice was significantly higher than that of the *β-arr1* KO mice following 5-FU treatment (Figure 6i). Our data suggest that *β*-arr1 has an important protective role in chemotherapy-induced CIGIS.

### *β*-arr1 reduced the chemotherapy-induced intestinal apoptosis by inhibiting ER stress-mediated mitochondrial apoptotic signaling

**Figure 7:**
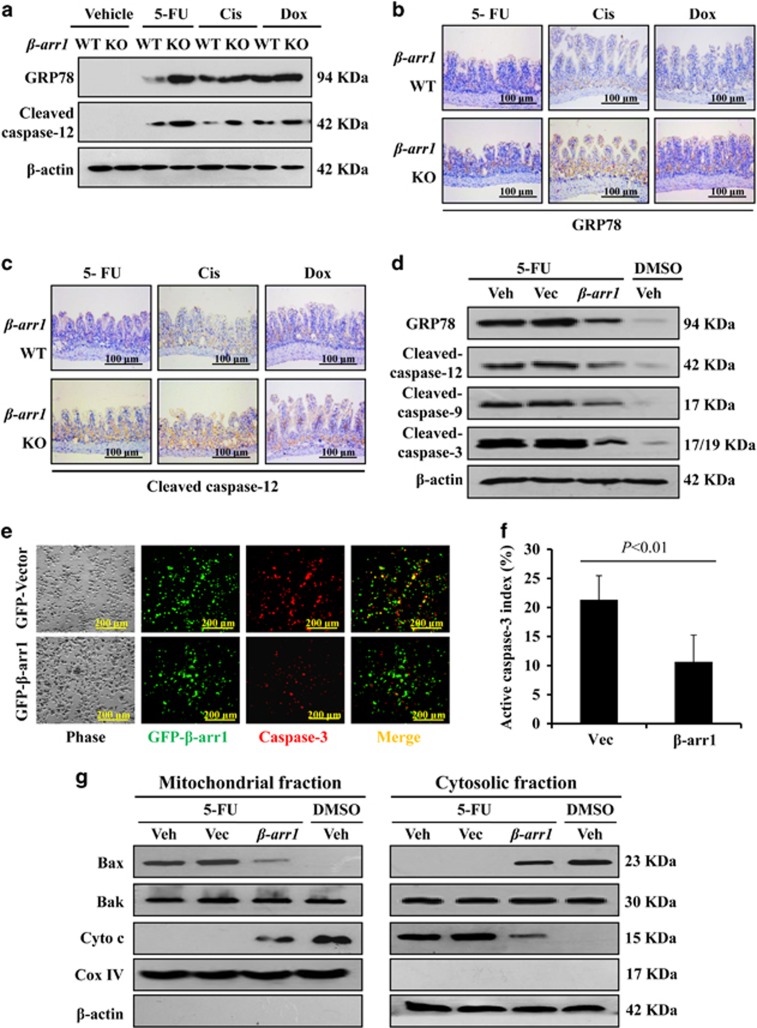
*β-arr1* ameliorated chemotherapy-induced intestinal apoptosis by downregulating ER stress-mediated mitochondrial apoptotic signaling. (**a**) Expression of GRP78 and cleaved caspase-12 protein was analyzed by western blotting, and *β*-actin was used as the loading control. Three independent experiments were performed. (**b**) Intestinal sections were stained for GRP78. (**c**) Intestinal sections were stained for cleaved caspase-12. (**d**) GRP78, caspase-12, caspase-9 and caspase-3 were examined in HCT116 cells following the indicated treatments by western blotting, and *β*-actin was used as the loading control. Veh, vehicle; Vec, vector. Three independent experiments were performed. (**e**) After 5-FU administration, HCT116 cells overexpressing *β*-arr1 were stained for caspase-3 (red). (**f**) The apoptotic index was determined in the cells following 5-FU administration. Values are shown as the mean±S.D. Three independent experiments were performed. (**g**) Bax, Bak and cytochrome *c* levels were determined in both mitochondrial and cytosolic fractions by western blotting, and *β*-actin and Cox IV were markers of cytosolic and mitochondrial fractions, respectively. Three independent experiments were performed

The apoptotic pathways have been linked to ER stress in recent studies.^17^ Thus, we determined whether ER stress has a key role in chemotherapy-induced intestinal apoptosis. The drugs 5-FU, Cis and Dox were administered to the mice, and both GRP78 and cleaved caspase-12 showed increased activation in *β-arr1* KO mice compared with WT mice (Figure 7a). Immunohistochemistry (IHC) staining confirmed that GRP78 and cleaved caspase-12 showed higher expression in *β-arr1* KO mice, especially in the intestinal crypts (Figures 7b and c). To further study the effect of *β*-arr1 in chemotherapy-induced intestinal apoptosis, a *β*-arr1 plasmid was transfected into HCT116 cells. Western blot analysis verified that *β*-arr1 overexpression downregulated the expression of GRP78 and inhibited the activation of caspase-12, caspase-9 and caspase-3 in 5-FU-induced apoptosis (Figure 7d). After cells were subjected to 5-FU for 12 h, *β*-arr1-overexpressing cells showed lower levels of apoptosis, and a drastic difference in apoptotic index was observed between *β*-arr1-transfected cells and vector-transfected cells (Figures 7e and f). These results revealed that *β*-arr1 alleviates intestinal apoptosis by downregulating ER stress after chemotherapy. In addition, 5-FU suppressed cytosolic accumulation of Bax and promoted mitochondrial cytochrome *c* release, which triggered mitochondrial apoptotic signaling. However, *β*-arr1 overexpression repressed cytochrome *c* release and inhibited 5-FU-induced mitochondrial apoptotic signaling (Figure 7g).

### *β-arr1* knockdown promoted chemotherapy-induced cell apoptosis via ER stress signaling

**Figure 8:**
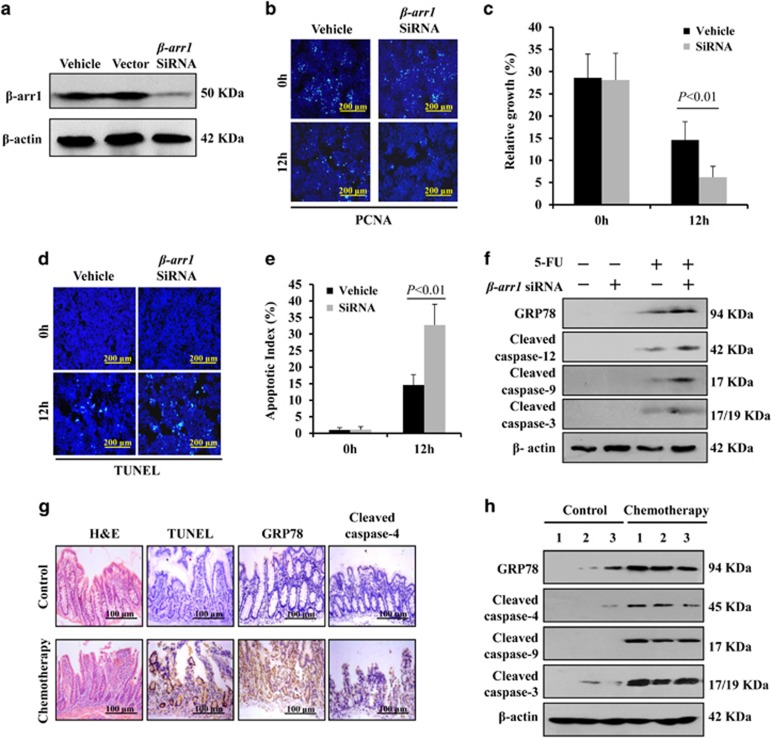
*β-arr1* knockdown promoted chemotherapy-induced apoptosis via ER stress signaling. (**a**) *β*-arr1 was examined in the indicated types of HCT116 cells by western blotting. (**b**) Cell proliferation was determined by PCNA staining (green) after 5-FU administration *in vitro*. (**c**) Cell growth at 12 h after 5-FU administration *in vitro.* PCNA-positive cells were counted in 20 different visual fields from each well. The relative growth (%) was determined by dividing the number of PCNA-positive cells by the total number of cells in 20 randomly selected visual fields. Values are shown as the mean±S.D. Three independent experiments were performed. (**d**) Cell apoptosis was analyzed by TUNEL staining (green) after 5-FU treatment *in vitro*. (**e**) TUNEL-positive cells were counted in four different visual fields from each well. The apoptotic index (%) was determined by dividing the number of apoptotic cells by the total number of cells in four randomly selected visual fields. Values are shown as the mean±S.D. Three independent experiments were performed. (**f**) ER stress-related proteins were evaluated by western blotting at 12 h following 5-FU treatment, and *β*-actin was used as an internal control. (**g**) The small intestine samples from patients in the control group and chemotherapy group were analyzed by H&E, TUNEL, GRP78 and cleaved caspase-4 staining, *n*=3 in each, and representative results are shown. (**h**) ER stress was activated in patients following chemotherapy. Three independent intestinal samples are shown

To confirm the protective effects of *β*-arr1 in CIGIS, we knocked down *β-arr1* expression using small interfering RNA (siRNA) in the HCT116 cell line (Figure 8a). Twelve hours after 5-FU administration, *β-arr1* siRNA markedly suppressed cell proliferation (Figures 8b and c) and induced cell apoptosis (Figures 8d and e) *in vitro.* Furthermore, consistent with the results *in vivo*, 5-FU increased GRP78, cleaved caspase-12, cleaved caspase-8 and cleaved caspase-3, which indicated the activation of ER stress, and knockdown of *β-arr1* further upregulated ER stress signaling following treatment with 5-FU (Figure 8f).

Finally, we examined intestinal mucosa changes and the signal pathways of CIGIS in humans. Accordingly, the intestinal mucositis, the apoptosis in the crypt and the activation of ER stress were also observed among chemotherapy patients (Figures 8g and h) following inhibition of *β*-arr1 after chemotherapy (Figure 4a). Our data indicated that *β*-arr1 inhibited chemotherapy-induced intestinal apoptosis via inhibiting ER stress signaling.

## Discussion

Despite the broad general knowledge regarding intestinal mucositis following cytotoxic damage,^18, 19^ there have been few direct assessments of the behavior of ISCs during chemotherapy. The potential impact on the crypt stem cells after chemotherapy has remained elusive.

Our study showed that chemotherapy induces small intestinal crypt cell apoptosis in humans and mice, specifically in Lgr5+ stem cells. This is consistent with our recent results using radiotherapy.^20^ Lgr5+ stem cell apoptosis was observed after exposure to three different chemotherapeutic drugs, 5-FU, Cis and Dox. Interestingly, Paneth cells also underwent apoptosis in this study. As Paneth cells are considered long-lived cells in the crypt,^21^ the mechanism underlying their apoptosis during CIGIS is still unclear and requires further study. Paneth cells constitute the niche for Lgr5+ stem cells in intestinal crypts and provide growth factor signals, such as epidermal growth factor (EGF), WNT3, and the NOTCH ligands DLL1 and DLL4.^22^ Genetic depletion of Paneth cells with Sox9 ablation led to Olfm4+ CBC stem cell loss.^22^ These cells are likely the major regulators of stem cell abundance through direct physical contact with the stem cells.^7^ Therefore, it is conceivable that the apoptosis of Paneth cells may lead to the decrease in Lgr5+ stem cells and the marked villus and crypt shrinkage, as well as the attenuation of proliferation. However, a recent study contradicted this possibility.^23^ After intestinal injury, Paneth cells were not required for intestinal regeneration, whereas Lgr5+ stem cells were indispensable.^23^ Thus, Lgr5+ stem cells have a primary role in CIGIS.

In addition to the small intestine, Lgr5+ stem cells have also been found in the hair follicles,^24, 25^ the stomach,^26^ the colon,^8^ colon cancer^27, 28, 29^ and rectal cancer.^29^ Thus, the behavior of these stem cells following chemotherapy may be similar in these tissues and contribute to the study of the chemotherapy-related side effects, including alopecia, vomiting and anorexia. This model may be used to investigate the curative effects of chemotherapy in GI malignancies by monitoring the Lgr5+ oncogenic stem cells.

Our previous study suggested that p53-mediated intestinal crypt cell apoptosis contributes to chemotherapy-induced intestinal mucosal injury.^5^ However, the upstream and downstream molecules in this apoptotic signaling pathway are still unknown. *β*-arr1, a GPCR, has enormous potential as a new therapeutic target in many diseases.^30^ Meanwhile, increasing evidence has indicated that *β*-arr1 mediated the activation of Mdm2 and subsequent degradation of p53;^13, 31^ thus, *β*-arr1 may affect CIGIS. Our recent study indicated an essential role for the GPCR pathway in the regulation of ER stress-mediated apoptosis.^6^ In this study, chemotherapy-induced intestinal apoptosis was increased at the bottom of the intestinal crypts in *β-arr1* KO mice, with activation of ER stress signaling. The results strongly indicated that *β*-arr1 serves as a linker between the GPCR and ER stress pathways. Lgr5+ stem cells have been reported to differentiate into different types of intestinal mucosal cells and are indispensable for intestinal regeneration.^6, 23, 32^ According to our study, the apoptosis after 5-FU treatment was predominantly found at positions 2–4 of the crypts in *β-arr1* KO mice, which is where Lgr5 is localized.^6^ Using double immunostaining, we also verified that Lgr5+ stem cells were more vulnerable to apoptosis in KO mice following chemotherapy. ER stress was associated with differentiation,^33^ and the degree of stem cell activity was affected by ER stress signaling.^7^ The Lgr5+ stem cells are highly sensitive to ER stress.^15^ All these data support our findings that chemotherapy suppressed *β*-arr1 to activate ER stress, and the ER stress induced Lgr5+ stem cell apoptosis, which resulted in CIGIS. The replacement and regeneration of Lgr5+ stem cell after chemotherapy and the role of *β*-arr1 and ER stress in these processes will be further studied.

CIGIS symptoms were much more severe in the *β-arr1* KO mice than those in the WT mice. The *β-arr1* gene increased survival in the CIGIS model, suggesting that *β*-arr1 effectively prevented CIGIS. These results provide a rationale to target *β*-arr1 in CIGIS for therapeutic purposes. Several factors, including EGF,^34^ insulin-like growth factor-1 (IGF-1)^35^ and R-spondin1,^36, 37^ have been shown to protect mice from chemotherapy-induced mucositis. EGF and IGF-1 are directly regulated by *β*-arr1. Thus far, no direct relationship between R-spondin1 and *β*-arr1 has been reported, but researchers found that R-spondin1 protected mice from chemotherapy-induced mucositis through the Wnt/beta-catenin pathway, and *β*-arrestin is a required component of Wnt/beta-catenin signaling *in vitro* and *in vivo*.^38, 39, 40, 41^ Thus, it is possible that *β*-arr1 affects R-spondin1 to mediate CIGIS protection. To date, the therapeutic potential of *β*-arrestin-based agents has been widely investigated,^9, 42, 43, 44^ but they have not been tested with intestinal disorders or CIGIS. Based on our results, we speculate that induction of *β*-arr1 expression (*β*-arr1-agonist) will be a potential therapy for CIGIS.

## Materials and Methods

### Human biopsy samples

Small intestinal biopsies were obtained from three cancer patients (two gastric cancers and one periampullary cancer) without small intestinal tumors who had received chemotherapy in the previous 2 weeks and manifested lower alimentary tract symptoms. Three patients were pooled in the chemotherapy group; two of them received the FOLFOX4 regimen, which included 5-FU, calcium folinate and oxaliplatin, and another was only administered oxaliplatin. The control group samples were obtained from three patients (one gastric cancer, one pancreatic cancer and one intestinal obstruction) without small intestinal disease who did not receive chemotherapy. During the operation, a sample from the small intestine (2–3 cm) was collected. All samples were acquired from The Third Affiliated Hospital of Sun Yat-sen University and Sun Yat-sen University Cancer Center. The acquisition of the tissue samples was approved by the Research Ethics Committee of The Third Affiliated Hospital of Sun Yat-Sen University. Written informed consent was obtained from each patient before inclusion in the study.

### Mouse genotyping and modeling

The C57BL/6 J mice, *β-arrestin-1*^*+/−*^ heterozygous mice with a C57B/L6 background (obtained from Dr. Robert J Lefkowitz, Duke University Medical Center, Durham, NC, USA), and *Lgr5-EGFP-IRES-creERT2*^*+/−*^ knock-in mice (Jackson Laboratory, Bar Harbor, ME, USA) were housed in the Sun Yat-sen University Vaccine Research Center. *β-arr1* WT and KO littermates were generated by breeding heterozygous mice. *Lgr5-EGFP-IRES-creERT2*^*+/−*^ knock-in littermates were generated by breeding *Lgr5-EGFP-IRES-creERT2*^*+/+*^ and *Lgr5-EGFP-IRES-creERT2*^*+/−*^ mice. *β-arr1*^*+/−*^ mice were crossed with *Lgr5-cre*^*+/−*^ mice, and *β-arr1*^*+/−*^*/Lgr5-cre*^*+/−*^ mice were generated. Furthermore, male and female *β-arr1*^*+/−*^*/Lgr5-cre*^*+/−*^ mice were bred to generate *β-arr1*^*+/+*^*/Lgr5-cre*^*+/−*^ and *β-arr1*^*−/−*^*/Lgr5-cre*^*+/−*^ mice.

The *β-arr1* mice were genotyped by real-time polymerase chain reaction (PCR) analysis using genomic DNA extracted from tail snips. *β-arr1* mutant primers are as follows: forward primer, 5′-CCTAGTGCTGGGATTACAAG-3′, and reverse primer, 5′-CATAGCCTGAAGAACGAGAT-3′ (obtained from Dr. Robert J Lefkowitz). The *Lgr5-EGFP-IRES-creERT2* mice were genotyped by PCR using genomic DNA extracted from tail snips. The WT reverse primer, 5′-ATACCCCATCCCTTTTGAGC-3′, and the mutant reverse primer, 5′-GAACTTCAGGGTCAGCTTGC-3′, were used. Sex-matched, 8- to 10-week-old mice (20–25 g) were used for all experiments with 8–10 mice in each group. CIGIS models were induced by i.p. injection of 5-FU (Sigma, St. Louis, MO, USA) at a dose of 75 mg/kg/day for 1, 3, 5 or 7 days. In addition, CIGIS models were also induced by i.p. injection of Cis (Sigma) at 5 mg/kg/day for 5 days and Dox (Sigma) at 6 mg/kg/day for 3 days.

### CIGIS assessment and survival rate

Mice were weighed once daily after beginning the 5-FU treatment. Five days later, mice were killed, and the colon was harvested. The number of well-formed stools was recorded. Similarly, animal well-being was assessed daily after the 5-FU (50 mg/kg/day) treatment, and the incidence and severity of diarrhea were scored. Scores were recorded as 0, 1, 2 or 3, where 0 is normal stool consistency, 1 is loose stools, 2 is overt diarrhea and 3 is liquid feces with severe perianal/tail soilage (extreme diarrhea).^45^

### Cells culture, transfection and induction of apoptosis

HCT116 cell lines were obtained from the American Type Culture Collection (ATCC, Manassas, VA, USA) and were routinely cultured in McCoy's 5A medium supplemented with 10% fetal bovine serum, 30 U/ml penicillin, and 30 mg/ml streptomycin at 37 °C under 5% CO_2_. HCT116 cells were transfected with a GFP-*β*-arr1 plasmid (kindly provided by Professor Pei G, Shanghai Institutes for Biological Sciences) using Lipofectamine 2000 (Invitrogen, Carlsbad, NM, USA). To assess chemotherapy-induced apoptosis, cells were incubated in culture medium containing 0, 10 or 100 *μ*g/ml 5-FU for 12 h. Cells were harvested and lysed in ice-cold sample buffer. Samples were stored for western blotting or fixed for microscopic assessment of apoptosis.

### Small interfering RNA

HCT116 cells were cultured in six-well plates. For *β-arr1* siRNA treatment, cells were transfected with 20 *μ*M *β-arr1* siRNA (Santa Cruz Biotechnology, Santa Cruz, CA, USA). Three *β*-arr1 siRNA sequences targeting the coding region of *β-arr1* mRNA were purchased, siRNA-1 (5′-GUCACCAACAACACCAACATT-3′), siRNA-2 (5′-GGGUCCUGUACAAUCUCAUTT-3′) and siRNA-3 (5′-CUGUACAUGUUUGGUUAAUTT-3′). The most effective sequence was selected. After incubation for 24 h, transfection medium was replaced by regular culture medium before 5-FU administration. A siRNA directed against glyceraldehyde-3-phosphate dehydrogenase provided in the kit was used as a control. After these cells had grown to a density of 90%, 10 *μ*g/ml of 5-FU was added for 12 h.

### Sample collection and preparation

Immediately after the animals were killed, the entire small intestine was carefully isolated and placed on ice. The 5 cm segments (duodenum) were removed. The rest of the intestine was rinsed thoroughly with ice-cold physiological saline as previously described.^46^

### Intestinal permeability and bacterial translocation

Inferior vena cava blood from the control group and the treatment group was collected. Blood DAO (mg/ml) and endotoxin (E*μ*/ml) levels were determined with an ELISA kit (Roche, Basel, Switzerland) to estimate intestinal permeability and bacterial translocation, respectively. ELISA was performed according to the manufacturer's instructions.

### Histological analysis and TUNEL staining

Formalin-fixed tissues were embedded in paraffin and sectioned. The 4 *μ*m sections were stained by hematoxylin and eosin (H&E). TUNEL staining was performed using the ApopTag kit (Roche) according to the manufacturer's instructions. The apoptotic index (%) was determined by dividing the number of apoptotic cells by the total number of cells in the epithelium of at least 20 randomly selected villi and crypts.

### PAS staining

Sections were dewaxed in xylene and rehydrated through a graded series of alcohols. Sections were oxidized in 1% periodic acid at room temperature for 20 min and rinsed in distilled water. Sections were treated for 30 min in Schiff's reagent (Sigma) and washed for 7 min in running water. Slides were counter-stained with methyl green, dehydrated and mounted.

### Antibodies and immunostaining

Primary antibodies used for immunostaining included those for EGFP (Santa Cruz Biotechnology), MMP7 (R & D Systems, Minneapolis, MN, USA), cytokeratin (Leica, Solms, Germany), CD34 (Abcam, Cambridge, MA, USA), GRP78 (Enzo Life Sciences, Lausen, Switzerland), cleaved caspase-3, cleaved caspase-4, cleaved caspase-9, cleaved caspase-12, Cox IV (all from Cell Signaling Technology, Danvers, MA, USA); secondary antibodies included goat anti-mouse HRP (Santa Cruz Biotechnology), goat anti-rabbit HRP (Santa Cruz Biotechnology), donkey anti-goat HRP (Santa Cruz Biotechnology), chicken anti-rat AP (Santa Cruz Biotechnology), chicken anti-rabbit AP (Santa Cruz Biotechnology), chicken anti-mouse AP (Santa Cruz Biotechnology), goat anti-rabbit Alexa 488/594 (Invitrogen), rabbit anti-goat Alexa 594 (Invitrogen), chicken anti-rabbit Alexa 594 (Invitrogen) and chicken anti-mouse Alexa 594 (Invitrogen). Immunostaining was performed using these antibodies. The sections were counter-stained with hematoxylin or nuclear fast red (Vector, Burlingame, CA, USA). For double staining, EGFP, MMP7, cytokeratin, CD34 and PAS staining was performed following TUNEL staining or cleaved caspase-3 staining.

### Antibodies and western blotting

Total protein was extracted, and mitochondrial and cytosolic fractions were analyzed by SDS-PAGE (Invitrogen) as previously described.^47^ The primary antibodies included those for p53 (Abcam), cleaved caspase-3 (Cell Signaling), Cox IV (Cell Signaling), Bax (Abcam), Bak (Abcam), cytochrome *c* (Santa Cruz Biotechnology), *β*-actin (Sigma) and *β*-arr1 (kindly provided by Dr. Robert J Lefkowitz, Duke University Medical Center). Appropriate horseradish peroxidase-conjugated secondary antibodies were used to detect the primary antibody/antigen complexes. ImageJ free software (http://imagej.nih.gov/ij/download.html) was used to quantify the results of western blotting.

### Statistical analysis

Quantitative data are expressed as the mean±S.D. Data were analyzed by an unpaired *t*-test or ANOVA, in which multiple comparisons were performed using the method of least significant difference. The survival rate was analyzed by the log-rank test. Statistical significance was defined as a *P*-value <0.05.

## Abbreviations

GI: gastrointestinal tract
CIGIS: chemotherapy-induced GI syndrome
*β*-arr1: *β*-arrestin1
ISCs: intestinal stem cells
5-FU: 5-fluorouracil
Cis: cisplatin
Dox: doxorubicin
CBC: crypt base columnar
WT: wild type
KO: knockout
DAO: diamine oxidase
IHC: immunohistochemistry
PAS: periodic acid–Schiff
siRNA: small interfering RNA
EGF: epidermal growth factor
IGF-1: insulin-like growth factor-1
